# Raised mammographic density: causative mechanisms and biological consequences

**DOI:** 10.1186/s13058-016-0701-9

**Published:** 2016-05-03

**Authors:** Michael J. Sherratt, James C. McConnell, Charles H. Streuli

**Affiliations:** Faculties of Life and Medical and Human Sciences, University of Manchester, Oxford Road, Manchester, M13 9PT UK

## Abstract

High mammographic density is the most important risk factor for breast cancer, after ageing. However, the composition, architecture, and mechanical properties of high X-ray density soft tissues, and the causative mechanisms resulting in different mammographic densities, are not well described. Moreover, it is not known how high breast density leads to increased susceptibility for cancer, or the extent to which it causes the genomic changes that characterise the disease. An understanding of these principals may lead to new diagnostic tools and therapeutic interventions.

## Background

The proportion of radio (X-ray)-opaque tissue within the breast is commonly referred to as mammographic density (MD). Epidemiologically, the risk of developing breast cancer is significantly greater in those women with raised MD. Whilst MD is therapeutically modifiable, patient tolerance to long-term endocrine treatments is low and the molecular and cellular causes of raised MD are, as yet, poorly understood. After briefly discussing breast anatomy, composition and density, this article reviews the current state of knowledge regarding the causative mechanisms of raised MD and the links between radio-opacity and cancer.

### Breast architecture and composition

Mammary gland architecture is reasonably simple, containing epithelial ‘trees’ that are surrounded by a connective tissue-rich stroma and interspersed with adipose tissue. Breast epithelium is composed of both spherical alveoli and a ductal network of tubes [1]. The alveolar epithelium is bilayered, containing apical luminal cells that make milk in lactation, and basal myoepithelial cells that contract around alveoli to squeeze milk into the ducts and thereby deliver it to the nipple. A continuous thin extracellular matrix (ECM) network of basement membrane surrounds all of the breast epithelium [2]. This provides instructive signals for epithelial cell behaviour, and also serves as a molecular barrier between the epithelium and the subtending stroma [3]. During ovarian cycles, breast epithelial cells undergo regular periods of proliferation and apoptosis [4]. These epithelial cells are the ones that can become mutated to cause breast cancer.

External to the ductal/lobular structure lies the stromal connective tissue. This provides a solid underpinning for the epithelium. It is constructed of fibroblastic cells that synthesise collagenous supportive ECM. The stroma is fairly thick around the ducts but much thinner around the secretory alveoli. Stromal-epithelial trees are surrounded by adipocytes to fill out the spaces, which together create the bag-like architecture of breast tissue. Amongst this cellular network also reside blood vessels, neuronal cells, and immune cells of various types. The overall composition is similar between different mammals, though there can be alterations in lobular architecture and the amount of stroma.

**Fig. 1:**
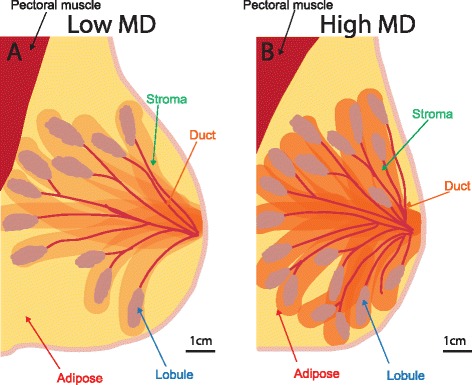
Diagrams of low versus high mammographic density (*MD*) breast. Tissue components and organisation within **a** low and **b** high MD breast. Stroma, ducts, lobules, and adipose are indicated. *Scale bar* = 1 cm

In the human breast, there are major differences in the extent of stromal compartments between different women. This is not normally seen within the genetically inbred strains of mice that are often used for studying mammary glands. However in outbred humans, the abundance of stromal tissue varies between small amounts with correspondingly large quantities of adipose, and high amounts that occupy a significant proportion of the breast. The combined stromal and epithelial component, in comparison to the total breast volume including adipose, is referred to as the percentage mammographic density (MD), and individuals have either high or low MD (Fig. 1).

### Breast cancer mechanisms

Breast cancer is a major disease that affects 12 % of the female population at some point during their lifetime, and is the global cause of death for nearly 500,000 women per year. It is caused in numerous ways. Inherited factors are responsible for about twice the overall population risk of getting breast cancer and some, such as mutated *BRCA* genes, cause 5–10 % of cases [5]. Histological and genomic analysis of tumours has revealed that there are several different types of breast cancer [6].

A crucial reason for identifying breast cancer-causing mechanisms is to provide early risk detection for patients, and thereby improved treatment. There have been tremendous advances in understanding the molecular basis of disease progression over the last 15 years. However, not very much is known about the mechanisms leading to the genomic changes that start breast cancer. Thus although oestrogen contributes in post-menopausal women, and overexpression of the *ErbB2* proto-oncogene is associated with cancer in around 30 % of cases [7], the other ways that breast epithelia are altered leading to the start of malignancy are not known.

Tissue architecture and ECM composition have central roles in controlling breast biology [2, 8, 9]. Moreover the biophysical characteristics of the tissue, which include X-ray density and mechanical stiffness, are of profound importance for breast biology and function [10]. However, these factors are highly variable between women.

**Fig. 2:**
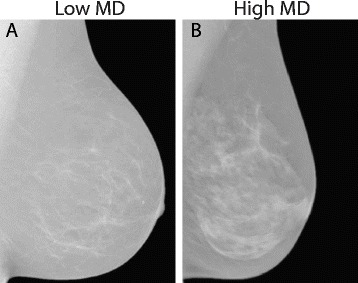
Mammograms of low versus high mammographic density (*MD*) breast. Mammograms of **a** low (Volpara-computed MD by volume = 3.0; age = 51 years) and **b** high (Volpara-computed MD = 18.5; age = 64 years) MD breast

Some biophysical properties of human breast tissue can be measured by mammography, a clinical practice that distinguishes between ‘high’- and ‘low’-density breasts [11]. Those with high MD contain a higher proportion of non-fatty tissue (Fig. 2). Importantly, there is a strong link between breast density and cancer, and mammography is now used widely for breast cancer screening. In the rest of this article we discuss the structural and compositional causes of different density, and the mechanistic links between high MD and cancer.

### Quantifying mammographic density

The emerging methods for assessing breast density were reviewed at the end of 2015 [12]. MD is commonly determined on a visual analogue scale (VAS) by a qualified radiologist; however, computational methods are increasingly used. Indeed, in post-menopausal women, there is a significant positive correlation between the percentage of dense breast tissue as measured by VAS, and that assessed by three-dimensional Volpara®, a commercial image analysis program (*r*^2^ = 0.695, *p* = 0.0001) [13–15].

MD is highly variable between women, ranging from a minimum of 3 % by volume, to very high levels of 25 % (Volpara® measurements; the latter high level is equivalent to 75 % density in VAS [16, 17]). These figures represent proportional differences in the amount of breast stroma/epithelia versus adipose. However, the molecular mechanisms causing these variations, and the manner in which the physical organisation of the ECM is mediated at microscopic length scales thereby determining MD, are not known.

### Clinical links between mammographic density and breast cancer

The current understanding of the link between MD and breast cancer was reviewed extensively in 2014, with over 180 of the previous papers on this topic examined [18]. Numerous studies, originating in 1976, have revealed that high MD is strongly linked with the susceptibility for breast cancer [19]. Indeed, women with high-dense breasts have a four- to sixfold greater risk of getting cancer than those with the lowest MD density. More recently, it was found that the percentage of high MD is a stronger risk factor for breast cancer than absolute dense area [20].

This density-cancer link is supported by studies in mice. For example, there is direct evidence from a mouse model where animals with a collagen I defect leading to stiffer ECM were crossed with those carrying a tumour virus oncogene [8]. In these animals, tumours form much more quickly, suggesting that collagen crosslinking rather than deposition (fibrosis) might promote tumorigenesis. In human breast disease, collagen crosslinking by lysyl oxidase enzymes such as lysyl oxidase-like 2 (LOXL2) promotes cancer risk as well as progression [21, 22]. Thus, in animal models, MD and ECM reorganisation contributes to breast cancer. However, in humans, the molecular basis linking MD and increased cancer risk remains unclear.

### Compositional and structural mediators of mammographic density

As yet, it is not known why MD varies between individuals. In some studies, body mass index (BMI) inversely correlates with high MD, possibly because increased adiposity reduces fibro-glandular components or stimulates stromal cells to differentiate into fat cells rather than to making collagen. Indeed, percentage dense area adjusted for BMI as well as age is a stronger risk factor then density alone [23]. However, not all studies concur. Although there is a link between BMI and cancer, there is no direct association between BMI and MD [13, 17]. Another potential cause of raised MD is the proliferation of ECM-producing stromal cells. In some, but not all, studies there is a link between raised MD and Ki-67 levels [24]. However, factors such as insulin-like growth factor-1 (IGF-1), which can be associated with proliferation, are not linked to MD.

**Fig. 3:**
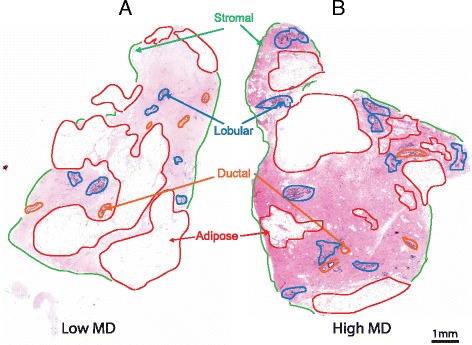
Histology of breast tissue composition. Paraffin section of **a** low and **b** high mammographic density (*MD*) breast, stained with hematoxylin and eosin, and imaged using conventional light microscopy. Stroma, ducts, lobules, and adipose are indicated. *Scale bar* = 1 mm

Regardless of the role played by cellular hypertrophy, an altered stromal composition in women aged 50–69 years correlates with increased MD, although there is no difference in the amount of epithelial lobules or ducts [25] (Fig. 3). In women with a high MD, there is an increase in the expression of small leucine rich proteoglycans, such as lumican and decorin, which may sequester extracellular growth factors, thereby promoting tissue remodelling. A large proteoglycan, versican, also accumulates in breast stroma, but only in X-ray dense areas where there is also malignant tumour progression particularly associated with micro-calcification [26]. Increased levels of collagen, TIMP-3, and IGF-1 are associated with high MD in women <50 years old [27]. However, in these cases the mechanism of altered production is not known. One possibility lies in macrophages, which can promote the formation of collagen fibrils [28].

It is also likely that fibroblasts are important mediators of tissue X-ray density and therefore MD, given their role in synthesising ECM components and their ability to differentiate into adipocytes. Isolated fibroblasts from high MD breasts accumulate less fat than those from low MD breasts, suggesting that fibroblast phenotype is different in high MD breast tissue [29]. Interestingly, CD-36, a collagen-binding thrombospondin receptor that is expressed on fibroblasts and promotes adipocyte differentiation, is often found at lower levels in the stroma of high MD individuals [30].

**Fig. 4:**
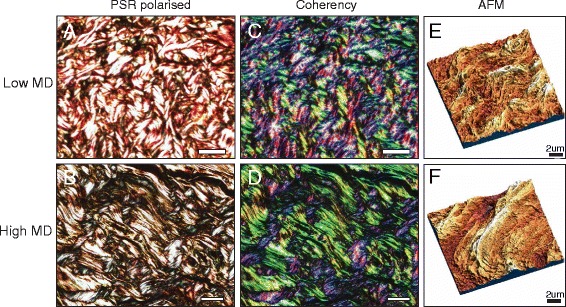
Fibrillar collagen organisation in low versus high mammographic density (*MD*) breast. Paraffin section of **a** low and **b** high MD breast, stained with picrosirius red (*PSR*) and visualised using polarised light microscopy. Both tissue samples contain abundant fibrillar collagen (*bright features*). *Scale bar* = 20 μm. Polarised light microscopy images of PSR-stained sections of **c** low and **d** high MD breast processed using the Image J plugin, OrientationJ. This applies a colour overlay to features of similar alignment, revealing fibril coherency. In this case, the high MD tissue contains many similarly aligned (*green*) features. *Scale bar* = 20 μm. Atomic force microscopy (*AFM*) topography images of regions within low **e** and high **f** MD breast. The organisation of collagen fibrils into large bundles in high MD tissues is evident over small length scales. *Scale bar* = 2 μm

Whilst there is some evidence for compositional remodelling as a mediator of raised MD, the effects of age and hormonal factors complicate interpretation of the data. We recently showed in an age-controlled cohort of post-menopausal women that MD may be influenced by structural remodelling of existing collagen fibrils rather than by collagen fibrosis [13]. Here, the architecture of high MD breast stromal tissue close to epithelial ducts changes, with the collagen I fibres becoming increasingly aligned and having greater coherency (Fig. 4). Although the periductal areas of breast stroma are significantly stiffer at cellular (micro-meter) length scales, both histological and mass spectrometry approaches failed to identify any change in local collagen concentration.

Studies using second harmonic generation imaging also reveal that collagen is more organised in high MD breasts [31]. The stroma may change within different regions of the normal breast. For example, interlobular breast stroma contains organised collagen fibrils and is stiffer than the ECM associated with peripheral regions of tissue containing endbuds [32]. However, although collagen architecture is different between high and low MD breasts, the mechanisms causing this remain obscure. Indeed, even the way that collagen fibres become orientated in the well-characterised and relatively simple tendon is still not known [33].

It seems likely that raised MD is associated with a complex pattern of upregulation and downregulation of ECM proteins. Mass spectrometry has revealed an extensive set of different ECM proteins within the mammary gland [34, 35]. By using this technique, we showed that high MD tissue contains significantly more periostin and collagen XVI than low MD breast. Periostin regulates collagen fibril morphology and potentially fibril crosslinking, and is often in tissues under high mechanical load [36]. It is overexpressed in most breast cancers, where it enhances angiogenesis and tumour progression [37]. Collagen XVI is an adapter protein that organises large fibrillar networks within the matrix, and its levels increase in inflamed tissue [38]. By promoting integrin signalling and cell/matrix binding, these proteins may induce cell proliferation and invasion [39]. Aberrant expression of ECM organising proteins may in turn cause architectural remodelling in the breast, stiffening of the local microenvironment, and promotion of a cancerous phenotype. However, more studies are required to better characterise the proteome of high MD stroma and to generate the corresponding genetic animal models.

### Genomic changes that characterise high mammographic density

High MD might promote genetic changes that cause tumorigenesis. Alternatively, it may reduce the levels of gene products that naturally prevent tumours from occurring. Thus, understanding the links between MD and genetic alterations is crucial. Several large genome-wide association studies (GWAS) have identified molecular markers that associate with high MD. Indeed, a study on twins suggested that at least some high MD genes might be inherited [40, 41]. Whilst GWAS analysis is not necessarily linked to alterations in breast tissue and does not reveal the process of forming MD, it provides a framework to explain how the genomic landscape of different density breasts change.

Single nucleotide polymorphisms are associated with both MD and breast cancer risk. These include: lymphocyte-specific protein 1 (*LSP1*), which binds f-actin and may be involved with cell migration); RAD51-like 1 (*RAD51L1*), which is involved in DNA repair and may sense DNA damage; XNF365; and chromosome 12q24 [42–44]. More recently, the density-associated genes have been extended to include: amphiregulin (*AREG*), an epidermal growth factor (EGF) family member strongly active in breast; *ESR1* (estrogen receptor); transmembrane protein 184B (*TMEM184B*), a receptor that can activate MAP kinase; megakaryoblastic leukemia 1 protein (*MKL1*), which transduces signals from the cytoskeleton to the nucleus, and may *trans*-activate serum response factor which is downstream of stiffness signals; PR domain containing 6 (*PRDM6*), a histone methyltransferase that may contribute to proliferation [45]; early B-cell Factor 1 (*EBF1*), a transcriptional activator; *MIR1972-2* (microRNA), netrin-4, a laminin-related ECM protein; myotubularin-related protein 11 (*MTMR11*), a pseudophosphatase that can be altered in some breast tumours; and Cezanne, which deubiquitinates EGF receptor and might also be involved in controlling high MD [46, 47]. *Tab2*, at the 6q25.1 locus, which binds ESR1 and is also involved with interleukin-1 activation of NFκB and MAPK8, is an additional genetic marker for high MD [48].

However, other inherited risk factors such as *BRCA1* and *BRCA2* are not linked to MD, and neither are breast cancer risk factors such as age at menarche, age of first child, and age of menopause, or parity, alcohol consumption, exercise, and family history [17, 49].

Together, these new studies reveal that MD shares a genetic component with breast cancer, and that some of the genes associated with it are involved with excess cellular proliferation. However, the extent to which these genomic studies are actually translated to changes at either the RNA or the protein level within breast tissue has not yet been established. One study has looked at RNA changes, though the findings are not conclusive [50]. Moreover, there are also most likely to be plenty of other, as yet unknown, genomic and consequent changes that also contribute risk. For example, aneuploidy rearrangements occur early in tumour evolution, and remain as tumours expand [51].

MD-associated genes are also different in oestrogen receptor *ER* change to *ER-positive* and *ER*+change to *ER-negative* patients, at least in the post-menopausal context [17]. Future genomic studies within breast tissue at the single cell level might therefore reveal novel gene rearrangements and identify mutations that contribute to the formation of high MD breast, and the tumours that result.

### Stromal changes in high-density breast

Changes in gene expression within the stromal compartment are also crucial. It is this part of the breast that contributes most to the local micro-stiffness that is perceived by epithelial cells. However, whilst it is clear that different breasts alter markedly in composition, structure, and mechanical properties, the molecular details of these variations remain poorly defined. To address these questions it would be valuable to apply next-generation sequencing, which has so far only been used to identify changes in normal breast mRNA levels at different stages of the menstrual cycle [52]. Similar studies comparing low and high MD breast tissue might reveal key expression changes of protein-encoding genes that occur within the stroma of high MD tissue. Hopefully, such genetic studies will collectively help to inform how different MDs are created.

### How altered connective tissue stiffness forms

Breast stiffness may subjectively be assessed via clinical palpation, but the mechanical properties of the tissue can also be measured. The most commonly used elastography approaches indirectly assess stiffness using ultrasound or magnetic resonance. Tissue stiffness can also be measured directly during mammography, by means of an array of pressure sensors coupled to the compression plate [53–55]. The relationship between tissue stiffness, MD, and breast cancer risk can also be explored by modelling approaches, where idealised assumptions about breast shape are used [10]. It is now clear that tissue stiffness varies both locally within the breast and between individuals. ECM assemblies play a key role in mediating the mechanical properties of tissues, and attention has focused primarily on collagens as the major stromal constituent.

In common with many connective tissues, breast stroma is enriched in fibrillar collagens. These collagens (primarily type I) are approximately three orders of magnitude stiffer than other ECM components such as elastin [56]. However, collagen deposition may not be the only, or indeed the major, driver of increased tissue stiffness in the breast. For example, it is clear from work in knockout mice that the organisation of collagen, in addition to its abundance, plays a key role in determining the mechanical properties of the tissue [57]. Decorin, a small leucine-rich proteoglycan which is found in normal breast tissue, coats the shaft of mature collagen fibrils preventing fibril fusion [58]. Whilst the skin of both wild-type and decorin knockout mice is rich in collagen fibrils, the abnormal morphology of fibrils in decorin-deficient tissue leads to increased skin fragility [57]. In human skin, chronic ultraviolet exposure is associated with the deposition of a disordered elastic-fibre protein-rich matrix, which is associated with significant skin stiffening [59, 60]. Although the mechanisms which drive ECM re-remodelling in the breast are poorly understood, matrix-organising proteins, such as periostin and collagen XVI, may therefore play a key role in mediating raised mammographic density [13].

In addition to deposition or re-organisation of the matrix, many long-lived proteins are prone to the accumulation of damage with age and disease [61]. In diabetes, for example, the normal slow accumulation of crosslinks that form advanced glycation end-products (AGEs) is accelerated by exposure to increased glucose concentrations. The aberrant glycation of proteins leading to AGEs is associated with increased tissue stiffness, and is also associated with breast cancer [62, 63]. Another potential driver of tissue stiffening is the accumulation of calcium, which is a feature of both benign and malignant breast lesions [64]. The role of these potential mechanisms in mediating X-ray absorption and local tissue stiffness, and thereby high MD, is a key question that will require further study.

### Mechanisms linking microenvironmental stiffness with cancer risk

As raised MD is a significant risk factor for cancer, it is important to understand the possible mechanisms leading to malignancy. There is a significant amount of work describing the influence of increased ECM stiffness on the promotion of later stages in breast cancer, for example as a mediator of cell migration and metastasis to form secondary tumours [65]. However, although breast tissue stiffness relates to cancer risk [10], virtually nothing is known about the link between increased local stiffness (at the microscopic length scales sensed by cells) and DNA damage, which is responsible for the earliest forms of disease.

Cells recognise a locally stiffened ECM via the detection and signalling machinery of integrins within multi-protein aggregates called adhesomes. Integrins are transmembrane receptors linking the outside microenvironment of a cell with its intracellular cytoskeletal structure and signalling. Altered ECM stiffness reorganises the architecture of the adhesome and its connection with the cytoskeleton, leading to profound changes in cell signalling and nuclear responses. One potential mechanism linking MD with cancer risk might involve signalling proteins that respond to different ECM stiffness. For example, focal adhesion kinase (FAK), p130Cas, and the Rho pathway are downstream targets of integrins that respond to modified cell-matrix interactions through integrin receptors. These, and other integrin-dependent proteins, promote proliferation in response to a stiff matrix, which in rare circumstances might lead to DNA damage and subsequent acquisition of mutant genotypes [66].

Another pathway induced by ECM stiffness involves Myc, which can increase the expression of the microRNA *miR18A*, leading to downregulation of *PTEN* and *HoxA9* [67]. High *miR18A* levels occur in clinical samples, and may predict luminal breast cancer. As well as possibly contributing to the genomic changes that cause the start of cancer, these pathways also increase cell migration and tumour invasion at later stages of the disease [68].

Downstream of ECM stiffness, one process that might lead to increased cancer risk is escape from apoptosis and the consequent DNA damage. Multiple signalling pathways are activated by mechanical stiffness, each of which impact on gene expression and may therefore promote cancer. For example, the YAP/TAZ pathway interprets mechano-chemical signals in the context of proliferation and organ size [69, 70]. Similarly, MRTF/SRF signalling acts downstream of tissue mechanics and the Rho pathway to control both the amount of actin assembled into mechano-sensitive stress fibres as well as downstream transcriptional targets [71]. The pathway is crucial in endothelial function, but less is known about its role in the breast [72].

Separate to conventional signalling pathways, direct links between the mechano-sensitive ECM and the nucleus include the Nesprin and Sun proteins. These connect the opposite ends of actin fibres from those at the plasma membrane with the nuclear membrane and chromatin [73]. In breast epithelia, altered external micromechanics via integrins can affect nuclear events, including, for example, the number of nucleoli, and may therefore influence longer-term cell fate decisions [74].

Although mechano-sensitive pathways that control cell fate in the short term are being uncovered, we do not yet know if any of these influence cancer risk in high MD breasts. If so, then possible longer-term therapeutic strategies might emerge. It will be important to recapitulate raised breast MD in mouse models, thereby enabling the role of mechano-sensitive signalling to be tested [75]. SCID mice might also provide new opportunities for growing human low and high MD breast samples, enabling an exploration of density-signalling mechanisms and therapeutic pathways to reverse it [76].

### Mammographic density as a marker to reduce the incidence of breast cancer

There are several therapeutic strategies to reduce or eliminate breast cancer from those who have it. The American Cancer Society sanctions six major kinds of treatment, including surgery, radiation therapy, chemotherapy, hormone therapy, targeted therapy, and bone-directed therapy. Whilst these are crucial, the introduction of novel biological therapeutic approaches is infrequent, and the main drug-based treatments remain as oestrogen blockers and aromatase inhibitors, and antagonists to receptor tyrosine kinases such as ErbB1/2. With high MD now being recognised as a major risk factor for cancer, the therapeutics that are used so far to reduce density and thereby to potentially protect against cancer arising from high MD mainly include selective ER modulators [77]. For example, the anti-oestrogenic compounds tamoxifen [78, 79], its relatives such as raloxifene [80] and aromatase inhibitors, are prescribed to patients with high MD but yet have no signs of acquiring breast cancer [81]. Interestingly, breast cancer-specific survival occurred in tamoxifen-treated women who showed a reduction in MD [82].

Whilst therapeutics have continued potential, one way of reducing the risk of breast cancer is to remove tumours as early as possible. This clinically tractable strategy considerably lowers disease risk in those individuals. A way forward will be to improve the identification of high MD breast in young women, for example those aged 44 or 47 years. In this way, those individuals can be assessed younger and more frequently, revealing early cancers for surgical removal. By understanding the molecular characteristics of high MD, it may also be possible to undertake screens that detect cancer earlier, thereby preparing medics and patients for drug treatment and surgery, and reducing breast cancer incidence. For example, a tumour-associated collagen signature-3 stain may be an indication of malignant breast carcinomas, and this could be extended by the use of markers newly identified within high MD tissues [83].

Of course, new therapeutics to reverse high MD will also add to this treatment arsenal. Better prognostic markers and therapies including altered diet [84], as well as a reduced use of post-menopausal oestrogen/progesterone therapy [85], might well contribute. Crucially, as suggested by our work, it may be the upregulation of ECM-organising proteins rather than structural ECM components themselves which cause increased MD and tissue stiffness. Proteins such as periostin, for example, which are regulated by microRNAs, may serve as potential therapeutic targets in the high MD breast [86]. Together, taking advantage of combined strategies to detect and/or revert high MD could reduce the incidence and mortality of breast cancer by more than 20 % [87].

## Conclusions

Raised MD is an important risk factor for breast cancer. Whilst considerable progress has been made in establishing both the potential mediators of breast X-ray density and the downstream biological consequences, many questions still remain. For example, it is not known why MD varies between healthy individuals or how high MD contributes to cancer risk. A key mediator of MD appears to be stromal abundance and architecture, and this differential tissue remodeling may be driven by biomechanical signaling feedback loops between the ECM-rich stroma and cells, or by intrinsic genetic differences. A greater understanding of these mechanisms is crucial for developing new therapeutic strategies to treat individuals with a raised risk of developing breast cancer.

## Abbreviations

AGE: advanced glycation end-product
BMI: body mass index
ECM: extracellular matrix
EGF: epidermal growth factor
ER: oestrogen receptor
GWAS: genome-wide association studies
IGF-1: insulin-like growth factor-1
MD: mammographic density
VAS: visual analogue scale
